# BiVO_4_/TiO_2_(N_2_) Nanotubes Heterojunction Photoanode for Highly Efficient Photoelectrocatalytic Applications

**DOI:** 10.1007/s40820-016-0115-3

**Published:** 2016-11-09

**Authors:** Rui Wang, Jing Bai, Yunpo Li, Qingyi Zeng, Jinhua Li, Baoxue Zhou

**Affiliations:** 1 School of Environmental Science and Engineering, Shanghai Jiao Tong University, Shanghai, 200240 People’s Republic of China; 2 Key Laboratory of Thin Film and Microfabrication Technology for Ministry of Education, Shanghai, 200240 People’s Republic of China

**Keywords:** BiVO_4_, TiO_2_(N_2_) nanotube, Heterojunction, Photoelectrocatalytic, Degradation of dyes

## Abstract

**Abstract:**

We report the development of a novel visible response BiVO_4_/TiO_2_(N_2_) nanotubes photoanode for photoelectrocatalytic applications. The nitrogen-treated TiO_2_ nanotube shows a high carrier concentration rate, thus resulting in a high efficient charge transportation and low electron–hole recombination in the TiO_2_–BiVO_4_. Therefore, the BiVO_4_/TiO_2_(N_2_) NTs photoanode enabled with a significantly enhanced photocurrent of 2.73 mA cm^−2^ (at 1 V vs. Ag/AgCl) and a degradation efficiency in the oxidation of dyes under visible light. Field emission scanning electron microscopy, X-ray diffractometry, energy-dispersive X-ray spectrometer, and UV–Vis absorption spectrum were conducted to characterize the photoanode and demonstrated the presence of both metal oxides as a junction composite.

**Graphical Abstract:**

Visible-light response BiVO_4_/TiO_2_(N_2_) naontubes photoelectrode was fabricated for photoelectrochemical water splitting and organic degradation in this paper. 
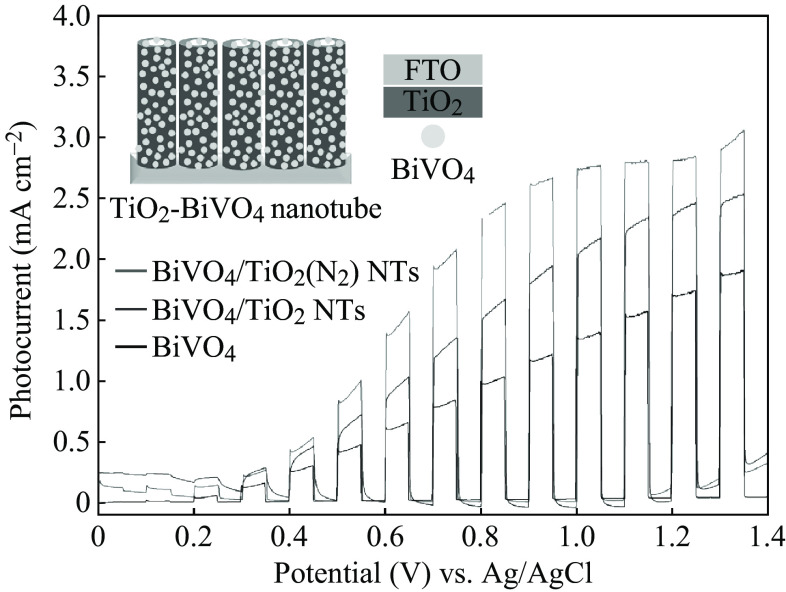

**Electronic supplementary material:**

The online version of this article (doi:10.1007/s40820-016-0115-3) contains supplementary material, which is available to authorized users.

## Introduction

The extreme shortage of natural resources and severe environmental problems caused by burning fossil fuels are pressing global concerns. In the past decades, many efforts were made to explore alternate energy sources. Photoelectrocatalytic (PEC) technology is widely recognized as an alternative energy source because it provides a highly efficient and eco-friendly route to produce renewable energy, and it degrades organic pollutants by the direct use of sunlight [1–4]. It can be achieved using a semiconductor photoanode/liquid junction, which drives an oxidation reaction. Therefore, in most PEC cells, the overall performance is primarily determined by the photoanode. However, it is still a challenge to synthesize a photoanode material that is chemically stable and has reasonably high incident light-to-current conversion efficiency in the visible range.

In recent years, Bi^3+^-based complex oxides that could absorb visible light effectively and with the advantage of price beneficial have been produced as alternative energy materials [5–8]. BiVO_4_ is a promising high efficient photoanode and photocatalysis material, with advantages of small optical band gaps (2.4 eV) and high stability, and low conduction band edges that overcome traditional photoanode materials, such as ZnO, TiO_2_, WO_3_, and Fe_2_O_3_ [9–13]. However, BiVO_4_ has the shortages of poor carrier transport properties and a substantially less efficient physical photoconversion rate [8].

One approach for alleviating these limitations is to use another semiconductor as support material to form a heterojunction that not only facilitates carrier transport but also enhances light absorption. Among various semiconductors, TiO_2_ has been intensively studied as a promising photoanode because it is stable, cost-effective, and has a negative flat band potential (∼0.2 V vs. RHE) (RHE, reversible hydrogen electrode) [14–18]. Recently, Xie et al. [19] found an unusual spatial transfer of visibly excited high-energy electrons of BiVO_4_ to TiO_2_, which indicated enhanced photoactivity in the heterojunction of BiVO_4_/TiO_2_ nanoparticles. Li et al. [20] demonstrated that a proper facet contact between BiVO_4_ and TiO_2_ nanoparticles was the key to improving the photoactivity of BiVO_4_. Recently, we studied one-dimensional (1D) nanostructured TiO_2_ coupled with a BiVO_4_ heterojunction with straight channels for electron transportation that reduced carrier diffusion lengths and improved charge collection efficiencies [21]. However, TiO_2_ has an intrinsically low mobility that limits the enhancement of photoactivity of the BiVO_4_–TiO_2_ heterojunction. Therefore, increasing the carrier concentration and also the conductivity in TiO_2_ is crucial to constructing a BiVO_4_–TiO_2_ heterojunction for a high-performance PEC cell.

In this study, we pre-treated TiO_2_ nanotubes in the nitrogen gas (TiO_2_(N_2_) NTs) and then coupled them with BiVO_4_ to form a BiVO_4_/TiO_2_(N_2_) NTs heterojunction. We find that the photocurrent is increased by approximately 30 % compared to those obtained by previously reported BiVO_4_/TiO_2_ NTs heterojunction [21]. Our PEC experiments further demonstrate the improved performance in the degradation of dyes. These results are attributed to the high carrier concentration of TiO_2_ NTs after annealing in a non-oxidizing atmosphere, as observed by Mott–Schottky spectra. In this case, the defects presented in the TiO_2_(N_2_) NTs increase the charge transfer kinetics, along with the reduced recombination losses due to trap filling. Thus, the charge transport between BiVO_4_ and TiO_2_ is enhanced to produce a higher photoactivity. This heterojunction provides useful insight into the design and fabrication of BiVO_4_-based photoanodes for potentially cost-effective and highly efficient PEC applications in large-scale applications.

## Experimental Procedures

### Preparation of BiVO_4_/TiO_2_(N_2_) NTs Photoanodes

TiO_2_ NTs were prepared by a template method in which ZnO nanowires (NWs) were transformed during a liquid-phase deposition (LPD) process. ZnO NWs were synthesized on FTO glass (2 × 2 cm^2^) after a hydrothermal treatment [22]. Next, a LPD treatment was conducted by placing ZnO NW substrates in a mixed solution of 50 mm (NH_4_)_2_TiF_6_ and 150 mm H_3_BO_3_ for 20 min at 25 °C [23]. After the LPD treatment, the sample was further annealed at 500 °C for 2 h in nitrogen gas, and nitrogen-treated TiO_2_ NTs were obtained and marked as TiO_2_(N_2_) NTs. For the fabrication of the BiVO_4_/TiO_2_(N_2_) NTs photoanode, a yellow precursor solutions of 300 mM Bi(NO_3_)_3_ and 300 mM NH_4_VO_3_ in 2 M HNO_3_ were deposited on the TiO_2_ NTs by spin coating [24]. Finally, the samples were sintered at 450 °C for 2 h in room air and yielded a yellow BiVO_4_/TiO_2_(N_2_) NTs film. For the control, the TiO_2_ NTs annealed in room air were used to prepare the BiVO_4_/TiO_2_ NTs photoanodes and bare BiVO_4_/FTO photoanodes were also prepared using the same procedure without the TiO_2_ NTs substrate.

### Structural Characterization

The morphologies of the samples were characterized using field emission scanning electron microscopy and a microscope equipped with an energy-dispersive X-ray spectrometer (EDX) (FEI, Sirion200) and TEM (JEM-2100F, JEOL, Japan). The crystalline phase of the samples was characterized by X-ray diffractometry (XRD) (AXS-8 Advance, Bruker, Germany). X-ray photoelectron spectroscopy (XPS) measurements were performed on an ESCALAB250 XPS measuring system with a Mg Kα X-ray source. Optical absorption measurements were conducted in a Lamda 750 UV–Vis–IR spectrophotometer using an integrating sphere.

### Photoelectrochemical Measurements

The photo responses of the BiVO_4_/TiO_2_ NTs photoanode were conducted using a three-electrode system with the Ag/AgCl electrode as the reference, platinum foil as the auxiliary electrode, and the samples as the working electrode. The working electrode potential and current were controlled by an electrochemical workstation (CHI 660c, CH Instruments Inc., TX, USA). A 350-W Xe lamp was used as a simulated light source, without further description, and all experiments were conducted under visible light (light intensity, 100 mW cm^−2^). The electrolyte was a 0.1 M Na_2_SO_4_ solution. The linear sweep voltammograms (LSV) were conducted under chopped light irradiation. The scan rate for the linear sweep voltammetry was 10 mV s^−1^. Photoluminescence (PL) measurements were conducted using an OmniPL-LF325 system with a 325 nm laser at room temperature. The incident photon-to-charge conversion efficiency (IPCE) was measured by a system comprising a monochromator (Zolix, P.R. China), a 500-W xenon arc lamp, a calibrated silicon photodetector, and a power meter. Mott–Schottky (impedance) spectra were recorded in 0.2 M Na_2_SO_4_ without light at a frequency of 1 kHz and a scan rate of 10 mV s^−1^.

Intensity modulated photocurrent spectroscopy (IMPs) was determined using an electrochemical workstation (ZENNIUM, ZAHNER-elecktrik GmbH & Co. KG, Germany) equipped with a controlled intensity modulated photospectroscopy setup (CIMPS, PP211, ZAHNER-elecktrik GmbH & Co. KG, Germany) after a two-electrode configuration. A white light lamp (WLC02, ZAHNER-elecktrik GmbH & Co. KG, Germany) was used as the light source. The modulated light in the frequency range of 0.1 Hz–1 kHz superimposed on a steady dc light with an intensity of 60 mW cm^−2^ was also used as a light source.

### Organics Compounds Degradation

The PEC degradation of the methylene blue (MB) experiment was conducted under the following conditions: visible light irradiation (100 mW cm^−2^), vigorous stirring, 1.0 V (vs. Ag/AgCl) of electric bias, pH 7, and 0.1 M sodium sulfate as the supporting electrolyte. Before degradation test, the nitrogen was bubbled to remove oxygen from the solution. The initial concentration of MB solution was 10 mg L^−1^ and the reaction solution was 20 mL during the experiment. The degradation rates of the dyes were analyzed with an UV–Vis spectrophotometer (UV2102 PCS, UNICO, Shanghai).

## Results and Discussion

**Fig. 1:**
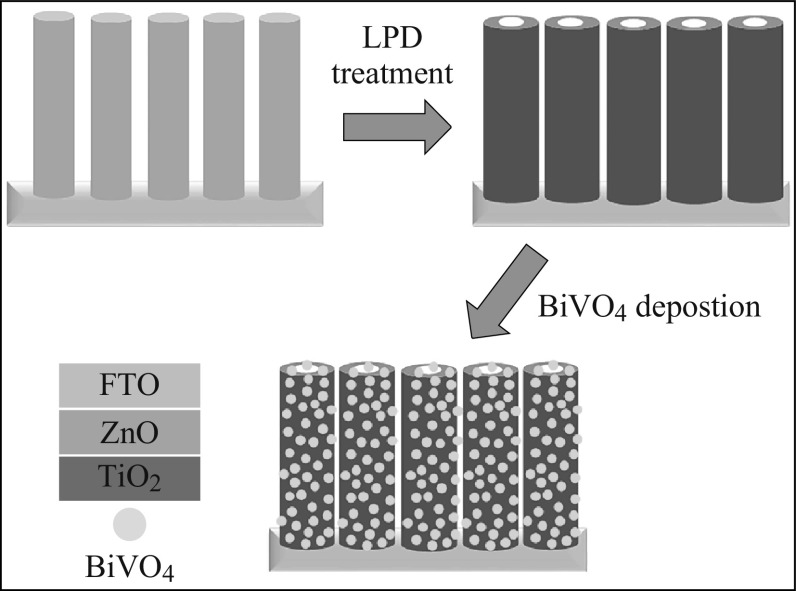
Schematic diagram of the main processes for the fabrication of the BiVO_4_/TiO_2_ NTs photoanodes

The main fabrication strategies for the BiVO_4_/TiO_2_(N_2_) NTs photoanodes are conducted in three steps as illustrated in Fig. 1. First, the ZnO NW template is grown on the FTO substrate through a hydrothermal method. Second, the template is transformed to TiO_2_ NTs after an LPD treatment which involves hydrolysis of ammonium hexafluorotitanate, and leads to the deposition of TiO_2_ as well as mild etching of ZnO from the formation of HF. Third, BiVO_4_ is deposited on the TiO_2_ NTs to form a photoactive composite layer.

**Fig. 2:**
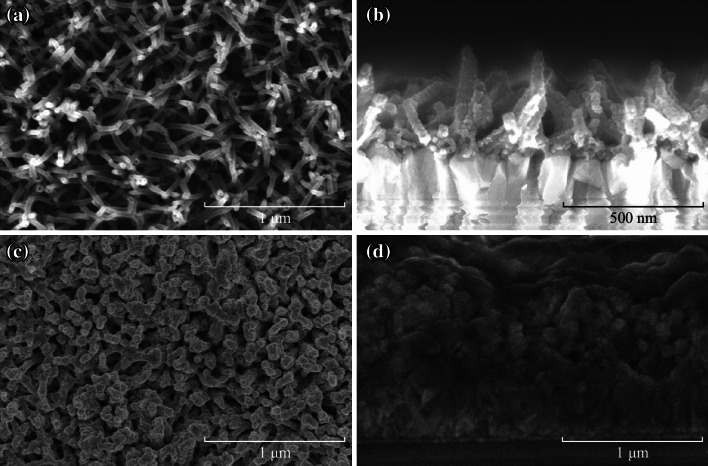
Top view and cross-sectional SEM images of TiO_2_(N_2_) NTs (**a**, **b**), and BiVO_4_/TiO_2_(N_2_) NTs (**c**, **d**)

Figure 2 shows the top and cross-sectional SEM images of optimized TiO_2_(N_2_) NTs and BiVO_4_/TiO_2_(N_2_) NTs, respectively. As shown in Fig. 2a, b, the obtained TiO_2_(N_2_) NTs have a vertical geometric shape, although the treatment of the NWs leads to partial connectivity among the constituent wires due to the surface tension during the evaporation of the solvent (Fig. 2a). Compared with the nitrogen-treated TiO_2_ NTs, the geometry for the air-annealed TiO_2_ NTs remains unchanged (not presented here). The TiO_2_ NTs are approximately 400 nm in length with a relatively rough surface (Fig. 2b). The top view SEM images of the BiVO_4_/TiO_2_(N_2_) NTs reveal that the TiO_2_(N_2_) NTs are completely covered by BiVO_4_ (Fig. 2c). Likewise, the side view also confirms the formation of the heterojunction of the BiVO_4_/TiO_2_(N_2_) NTs heterojunction (Fig. 2d). The thickness of the junction is approximately 600 nm, which is thicker than that of pure BiVO_4_ photoanode (Fig. S1). As shown in Fig. S2, the TEM images also demonstrate the heterojunction structure, where the BiVO_4_ nanoparticles are clearly observed on the TiO_2_ NTs.

**Fig. 3:**
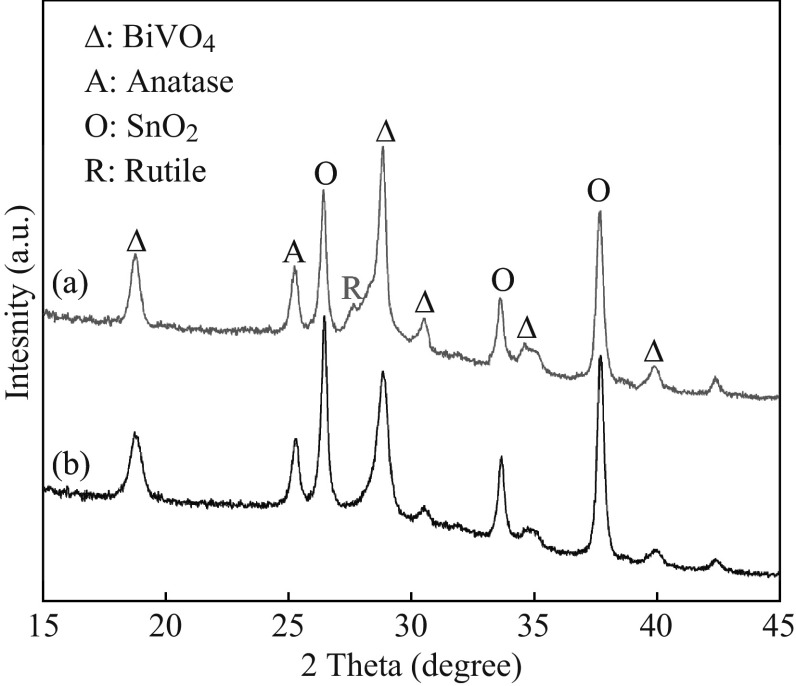
XRD patterns of **a** BiVO_4_/TiO_2_ NT and **b** the BiVO_4_/TiO_2_(N_2_) NTs electrode

**Fig. 4:**
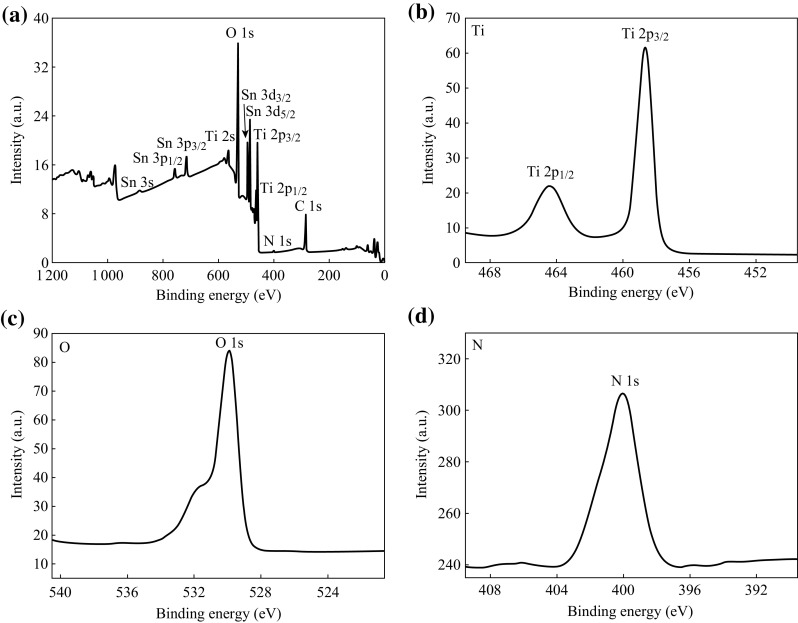
**a** X-ray photoelectron spectroscopy survey scan over a large energy range at low-resolution and high-resolution Ti 2p, **b** O 1s, **c** N 1s, **d** XPS spectra for TiO_2_(N_2_) NTs

The elemental composition of the BiVO_4_/TiO_2_(N_2_) NTs was also analyzed and their characteristic elements were identified using an EDX detection spectrometer. As shown in Fig. S2, the elements of Bi and V have almost the same percentage of atoms (%), indicating the formation of BiVO_4_. XRD also measured the crystalline phases of BiVO_4_ and BiVO_4_/TiO_2_ NTs, and the results are shown in Fig. 3. For all samples, the prominent peaks for BiVO_4_ are likely derived from the monoclinic phase of BiVO_4_ (PDF 14-0688). The typical peaks at 25.3° and 27.4° are assigned to the (101) and (110) planes of anatase and rutile phases, respectively. In Fig. 3a, the annealed composite has anatase phase and a large amount of rutile phase from the integrated intensity of the peaks associated with the (101) and (110) planes. However, for the BiVO_4_/TiO_2_(N_2_) NTs sample, it contains mostly anatase (Fig. 3b). These results are in accord with the reports by Jin et al. [25] and Mahajan et al. [26], who studied the effects of the atmosphere on the crystalline phase of TiO_2_ nanotube arrays in the annealing process. Also, the peaks at 26.4° and 37.6° for both samples are ascribed to the FTO substrate. To further study the surface composition and chemical state of TiO_2_(N_2_), XPS analysis was also conducted, and the results are illustrated in Fig. 4. The full survey indicates the presence of Sn, O, Ti, and N (Fig. 4a). Figure 4b–d shows the high-resolution XPS spectra of the elements, respectively. For the O 1s (Fig. 4b), the peak at 531.0 eV corresponds to the lattice oxygen, which is related to the Ti–O or Sn–O chemical bonding in the SnO_2_ or TiO_2_. Two distinct peaks located at 464.5 and 458.7 eV in Fig. 4c are assigned to the binding energy of Ti 2p_1/2_ and Ti 2p_3/2_, respectively, indicating the presence of Ti^3+^. The peak at 400.1 eV could ascribe to γ-N state, which is molecularly chemisorbed N_2_ [27].

**Fig. 5:**
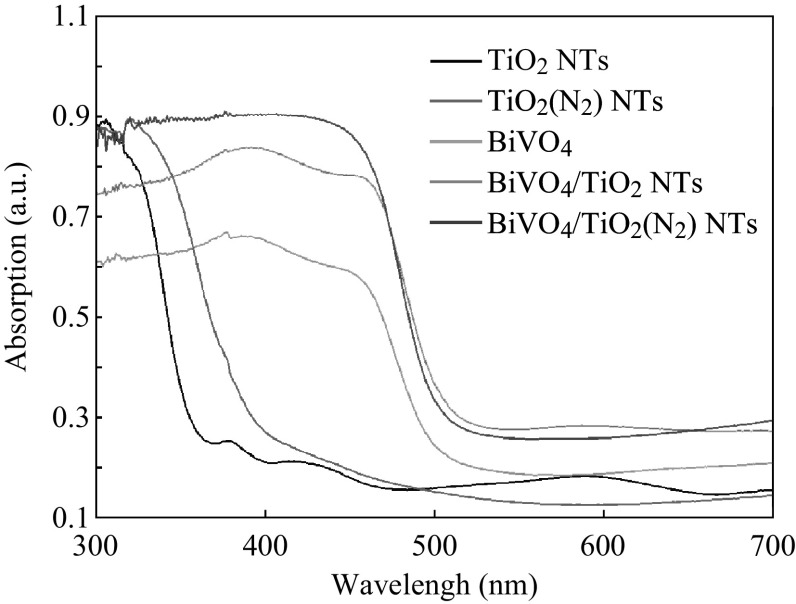
Photo-absorption spectra of the TiO_2_ NTs, TiO_2_(N_2_) NTs, BiVO_4_, BiVO_4_/TiO_2_ NTs, and the BiVO_4_/TiO_2_(N_2_) NTs, respectively

The optical absorption spectra of the TiO_2_ NTs, TiO_2_(N_2_) NTs, BiVO_4_/TiO_2_ NTs, and the BiVO_4_/TiO_2_(N_2_) NTs are shown in Fig. 5. The TiO_2_ NTs show an absorption edge at ~360 nm, whereas, the TiO_2_(N_2_) NTs with an absorption tails extend into the visible wavelength regions. The long absorption tail indicates the presence of additional energy states within the band gap of TiO_2_. The energy may have resulted from the presence of oxygen vacancies or non-stoichiometric TiO_2_ due to annealing in a non-oxidizing atmosphere. On the other hand, the pure BiVO_4_ film displayed absorption within the visible region of the spectrum with the edge at ~516 nm, which corresponded to the band gap energy of 2.4 eV and further demonstrated the formation of monoclinic phase BiVO_4_ [28]. After the deposition of BiVO_4_, both the BiVO_4_/TiO_2_ NTs and the BiVO_4_/TiO_2_(N_2_) NTs had very similar band gap absorption compared to BiVO_4_, although they had enhanced intensities in the visible region. The enhanced absorption intensity was attributed to the thicker BiVO_4_ film in the heterojunction as observed in the SEM images.

**Fig. 6:**
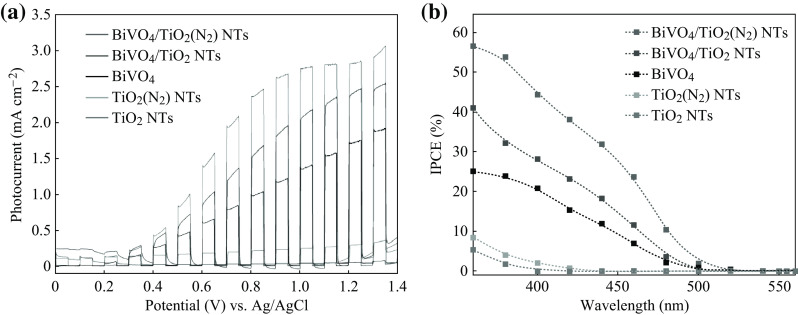
**a** Photoelectrochemical responses of the TiO_2_ NTs, TiO_2_(N_2_) NTs, BiVO_4_, BiVO_4_/TiO_2_ NTs, and the BiVO_4_/TiO_2_(N_2_) NTs under illumination of chopped visible irradiation in 0.1 M Na_2_SO_4_ solution and **b** corresponding IPCE spectra

Figure 6 presents the LSV characteristics of the TiO_2_ NTs, TiO_2_(N_2_) NTs, BiVO_4_,BiVO_4_/TiO_2_NTs, and the BiVO_4_/TiO_2_(N_2_)NTs, respectively. The TiO_2_ NTs sample exhibited a pretty low photocurrent under visible irradiation due to its large band gap, whereas the TiO_2_(N_2_) NTs sample had a slight photocurrent. The photocurrent for pure BiVO_4_ increased steadily with the increasing potential of the working electrode, and a photocurrent density of 1.36 mA cm^−2^ (1.0 V vs. Ag/AgCl) was obtained. Compared to that of pure BiVO_4_, a significant enhancement in photocurrent, ca. 2.06 mA cm^−2^ (1.0 V vs. Ag/AgCl), by the BiVO_4_/TiO_2_ NTs was observed. The photocurrent was further enhanced by approximately 30 % when using the BiVO_4_/TiO_2_(N_2_) NTs, which obtained the photocurrent of 2.73 mA cm^−2^ (1.0 V vs. Ag/AgCl). The BiVO_4_/TiO_2_(N_2_) with the cyclic voltammetry test also shows a stable photocurrent in the measuring range (Fig. S4).

Incident photon-to-current efficiency was measured in order to ascertain the light conversion efficiency of heterojunction of the BiVO_4_/TiO_2_(N_2_) NTs and was compared to the BiVO_4_/TiO_2_ NTs, BiVO_4_, and TiO_2_ in Fig. 6b. Due to a large band gap, both the TiO_2_ NTs and TiO_2_(N_2_) NTs had low efficiencies below 400 nm, although the TiO_2_(N_2_) NTs exhibited better performances. The IPCE of BiVO_4_ was comparatively at ~20 % at 410 nm, whereas heterojunction BiVO_4_/TiO_2_ NTs had a higher IPCE at nearly 28 % at 410 nm. Comparably, the IPCE of BiVO_4_/TiO_2_(N_2_) NTs further increased to 44 % at 410 nm, which was more than 100 % higher than the IPCE of bare BiVO_4_. This again suggests that the rectifying electron transfer from BiVO_4_ to TiO_2_ likely inhibits the fast recombination and increases the solar energy conversion efficiency of the junction. The IPCE was nearly zero at 550 nm, which is consistent with the optical absorption of the samples.

**Fig. 7:**
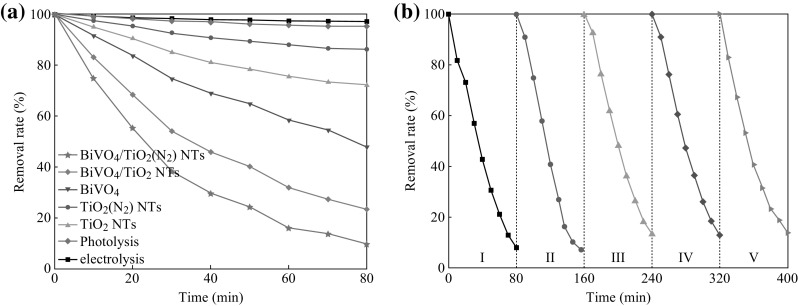
**a** PEC degradation of MB using different photoanodes under visible light illumination and **b** stability of BiVO_4_/TiO_2_(N_2_) NTs photoanodes for degradation of MB during a series of five identical tests

The PEC properties of the BiVO_4_/TiO_2_(N_2_) NTs were investigated by treating the organic dye (MB) under visible light illumination. It can be seen that almost no MB or little MB can be directly degraded by only applying electrocatalytic or photolytic reaction, and the TiO_2_ NTs only resulted in a removal ratio of only 14.1 % within 80 min, whereas the TiO_2_(N_2_) NTs had a higher efficiency of 27.2 % under the same conditions. The limited improvement in degradation of MB by TiO_2_ NTs was due to a large band gap that limited the use of visible light. Compared to the TiO_2_ NTs, the BiVO_4_ electrode degraded 52.4 % of the MB within the same time because of good absorption in the visible region. For the BiVO_4_/TiO_2_ NTs, the removal rate increased to 76.7 % due to fast electron transfers between the BiVO_4_ and TiO_2_ NTs. However, it is easily observed from Fig. 7a that the BiVO_4_/TiO_2_(N_2_) NTs obtained the removal rate of 91.8 % under the same conditions. The recycle performance of the BiVO_4_/TiO_2_(N_2_) NTs for PEC degradation of MB was investigated in five PEC cycles, and the results are shown in Fig. 7b. These results further suggested that the BiVO_4_/TiO_2_ NTs were stable for PEC applications, such as treating organic wastewater [29–31]. During all the process in PEC, we use 1 cm^2^ photoanode under visible light illumination to react.

**Fig. 8:**
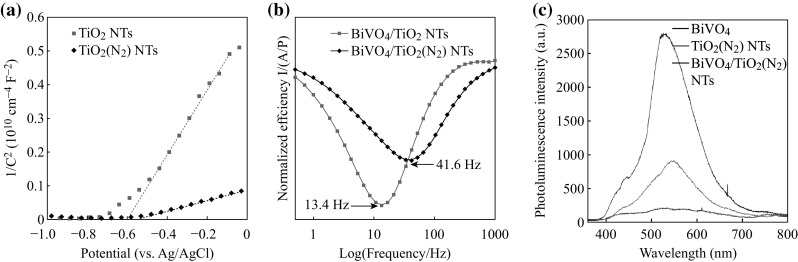
**a** Mott–Schottky plots for the TiO_2_ NTs and TiO_2_(N_2_) NTs electrodes measured in 0.2 M Na_2_SO_4_ at 1 kHz and **b** IMPS for BiVO_4_/TiO_2_ NTs and BiVO_4_/TiO_2_(N_2_) NTs, **c** PL spectra for BiVO_4_, TiO_2_(N_2_) NTs, and BiVO_4_/TiO_2_(N_2_) NTs

As previously discussed, the BiVO_4_/TiO_2_(N_2_) NTs exhibited a significant enhancement in photoactivity as verified by higher photocurrent as well as a higher PEC efficiency in the degradation of dyes. Apparently, the TiO_2_(N_2_) NTs played an important role in the promotion of the charge transfers in the electrode. We concluded that the carrier concentration in the TiO_2_ NTs could be increased after annealing in a nitrogen atmosphere. To make sure the impacts of the TiO_2_(N_2_) NTs, impedance measurements were carried out at a frequency of 1 kHz on both the TiO_2_(N_2_) NTs and TiO_2_ NTs electrodes in 0.2 M Na_2_SO_4_ electrolytes in the dark. The results are demonstrated by the Mott–Schottky plots in Fig. 8a. From the linear portion of the Mott–Schottky plots, charge carrier densities are calculated using the relation

1 $$N_{\text{D}} = \frac{2}{{e\varepsilon \varepsilon_{0} m^{\prime}}}$$

where *N*_D_ is the charge carrier density, *e* is the elementary electron charge (*e* = 1.6 × 10^−19^ C), *ε* is the dielectric constant (*ε* = 48), *ε*_0_ is the permittivity in vacuum (*ε*_0_ = 8.85 × 10^−12^ F m^−1^), and *m* is the slope of the 1/C^2^ versus potential plot. A charge carrier density of 2.9 × 10^18^ cm^−3^ was determined for the TiO_2_ NTs, but was 2.1 × 10^19^ cm^−3^ for the TiO_2_(N_2_) NTs. These results indicated that the charge carrier concentration of the TiO_2_ NTs was indeed increased after calcination in the non-oxidizing atmospheres. The higher defect density of the nitrogen-annealed sample also involved a higher electrical conductivity [32] and rapid charge transfer.

To further confirm enhanced charge transfers between BiVO_4_ and the TiO_2_(N_2_) NTs in the heterojunction material, the transit time (*τ*_d_) of the majority carriers in the BiVO_4_/TiO_2_ NTs electrode and the BiVO_4_/TiO_2_(N_2_) NTs electrode was measured by IMPS, respectively. The transit time *τ*_d_ was the average time that the photogenerated charges took to transfer to the back contact, and were estimated from the equation *τ*_d_ = (2π·*f*_min_ (IMPS))^−1^, where *f*_min_ is the frequency at the minimal value in the IMPS plot. The transit time reflects the recombination probability of the photogenerated electrons and holes in the photoelectrode [33]. Figure 8b shows the IMPS plots of the BiVO_4_/TiO_2_ NTs electrode and the BiVO_4_/TiO_2_(N_2_) NTs electrode, respectively. According to the previous equation, the transit time *τ*_d_ for the BiVO_4_/TiO_2_ NTs was 11.9, and 3.82 ms for BiVO_4_/TiO_2_(N_2_) NTs electrode, which indicated that the transport speed of the majority of photogenerated charges in the BiVO_4_/TiO_2_(N_2_) NTs electrode was three times faster than that of the BiVO_4_/TiO_2_ electrode. In other words, the BiVO_4_/TiO_2_(N_2_) NTs heterojunction could facilitate the majority of the photogenerated charges transported to the counter electrode and likewise, the transport of photogenerated electrons to the electrolyte is enhanced.

The transportation of electrons between the two materials was also certified by PL measurement as shown in Fig. 8c. We observed strong emission from bare TiO_2_ NTs and BiVO_4_, whereas the BiVO_4_/TiO_2_ heterojunction resulted in a near 90 % reduction in the emission intensity. The obvious quenching of luminescence of BiVO_4_ is characteristic of charge transfer between the BiVO_4_ and TiO_2_ NTs, implying a strong indication of the efficient reduction in recombination of charge carriers in the 1D heterojunction material. In consequence, the separation efficiency of photogenerated electron–hole pairs in BiVO_4_/TiO_2_(N_2_) NTs heterojunction could be improved.

**Fig. 9:**
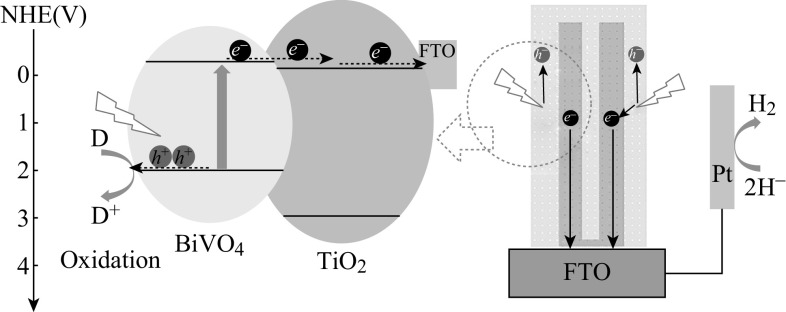
Schematic of energy bands and charge transfers at BiVO_4_/TiO_2_(N_2_) NTs photoanodes

Based on the experiments, We concluded that the improved performance of the BiVO_4_/TiO_2_(N_2_) NTs was primarily due to enhanced optical absorption and specific TiO_2_(N_2_) NTs. The nanotube structure provides larger surface area than the planar structure so that more BiVO_4_ photocatalyst was loaded for absorbing more visible light. On the other hand, the presence of oxygen vacancies or non-stoichiometric TiO_2_ in the TiO_2_(N_2_) NTs significantly enhanced the carrier density which favors the separation of photo-introduced electron–hole pairs verified by IMPS test. Thus, the higher photocurrent was obtained. The whole PEC system is shown in Fig. 9. Upon excitation by visible light, electrons were photoexcited from the valence band of BiVO_4_ to its conduction band. Then electron differences in the positions of the conduction bands which drove to photoelectrons generated in BiVO_4_ to the tubular TiO_2_(N_2_) NTs, where electrons were rapidly separated and directed to the Pt counter electrode via the external circuit. Consequently, the photogenerated electrons were scavenged by hydrogen ions on the Pt foil, and formed hydrogen gas, while the photogenerated holes oxidized water or organics on the surface of the BiVO_4_. Overall, the BiVO_4_/TiO_2_(N_2_) NTs heterojunction offered remarkable photoconversion efficiency.

## Conclusions

A visible light response BiVO_4_/TiO_2_(N_2_) NTs photoelectrode was fabricated for photoelectrochemical (PEC) organic degradation. Mott–Schottky plots and IMPS demonstrated the increased carrier concentration in the TiO_2_(N_2_) NTs, which enhanced electron transfers between BiVO_4_ and TiO_2_. A photoelectrochemical measurement confirmed that the photocurrent was increased approximately 100 % using the heterojunction when compared to bare BiVO_4_ under 100 mW cm^−2^ visible light illumination. Due to its excellent photoactivity and stability, the BiVO_4_/TiO_2_(N_2_) NTs show a promising future in PEC applications.

## Electronic Supplementary Material

Below is the link to the electronic supplementary material.

Supplementary material 1 (DOCX 1529 kb)

## References

[1] Walter MG, Warren EL, McKone JR, Boettcher SW, Mi QX, Santori EA, Lewis NS (2010). Solar water splitting cells. Chem. Rev..

[2] Bai J, Li JH, Liu YB, Zhou BX, Cai WM (2010). A new glass substrate photoelectrocatalytic electrode for efficient visible-light hydrogen production: CdS sensitized TiO_2_ nanotube arrays. Appl. Catal. B.

[3] Liu Z, Zhang X, Nishimoto S, Jin M, Tryk DA, Murakami T, Fujishima A (2008). Highly ordered TiO_2_ nanotube arrays with controllable length for photoelectrocatalytic degradation of phenol. J. Phys. Chem. C.

[4] Bai J, Liu YB, Li JH, Zhou BX, Zheng Q, Cal WM (2010). A novel thin-layer photoelectrocatalytic (PEC) reactor with double-faced titania nanotube arrays electrode for effective degradation of tetracycline. Appl. Catal. B.

[5] Tang JW, Zou ZG, Ye JH (2004). Efficient photocatalytic decomposition of organic contaminants over CaBi_2_O_4_ under visible-light irradiation. Angew. Chem. Int. Ed..

[6] Zou ZG, Arakawa H (2003). Direct water splitting into H_2_ and O_2_ under visible light irradiation with a new series of mixed oxide semiconductor photocatalysts. J. Photochem. Photobiol. A.

[7] Kudo A (2003). Photocatalyst materials for water splitting. Catal. Surv. Asia.

[8] Park Y, McDonald KJ, Choi KS (2013). Progress in bismuth vanadate photoanodes for use in solar water oxidation. Chem. Soc. Rev..

[9] Kuang YB, Jia QX, Nishiyama H, Yamada T, Kudo A, Domen K (2016). A front-illuminated nanostructured transparent BiVO_4_ photoanode for >2% efficient water splitting. Adv. Energy Mater..

[10] Thalluri SM, Hernandez S, Bensaid S, Saracco G, Russo N (2016). Green-synthesized W- and Mo-doped BiVO4 oriented along the 040 facet with enhanced activity for the sun-driven water oxidation. Appl. Catal. B.

[11] Kim TW, Choi KS (2014). Nanoporous BiVO_4_ photoanodes with dual-layer oxygen evolution catalysts for solar water splitting. Science.

[12] Zhang L, Chen DR, Jiao XL (2006). Monoclinic structured BiVO_4_ nanosheets: hydrothermal preparation, formation mechanism, and coloristic and photocatalytic properties. J. Phys. Chem. B.

[13] Hu Y, Li DZ, Zheng Y, Chen W, He YH, Shao Y, Fu XZ, Xiao GC (2011). BiVO_4_/TiO_2_ nanocrystalline heterostructure: a wide spectrum responsive photocatalyst towards the highly efficient decomposition of gaseous benzene. Appl. Catal. B.

[14] Bai J, Zhou BX (2014). Titanium dioxide nanomaterials for sensor applications. Chem. Rev..

[15] Lan XZ, Bai J, Masala S, Thon SM, Ren Y (2013). Self-Assembled, nanowire network electrodes for depleted bulk heterojunction solar cells. Adv. Mater..

[16] Gao B, Kim YJ, Chakraborty AK, Lee WI (2008). Efficient decomposition of organic compounds with FeTiO_3_/TiO_2_ heterojunction under visible light irradiation. Appl. Catal. B.

[17] Sridharan K, Jang E, Park TJ (2013). Novel visible light active graphitic C_3_N_4_–TiO_2_ composite photocatalyst: synergistic synthesis, growth and photocatalytic treatment of hazardous pollutants. Appl. Catal. B.

[18] Liu Y, Zhou H, Li J, Chen H, Li D, Zhou B, Cai W (2010). Enhanced photoelectrochemical properties of Cu_2_O-loaded short TiO_2_ nanotube array electrode prepared by sonoelectrochemical deposition. Nano-Micro Lett..

[19] Xie MZ, Fu XD, Jing LQ, Luan P, Feng YJ, Fu HG (2014). Long-lived, visible-light-excited charge carriers of TiO_2_/BiVO_4_ nanocomposites and their unexpected photoactivity for water splitting. Adv. Energy Mater..

[20] Li HF, Yu HT, Quan X, Chen S, Zhao HM (2015). Improved photocatalytic performance of heterojunction by controlling the contact facet: high electron transfer capacity between TiO_2_ and the 110 facet of BiVO_4_ caused by suitable energy band alignment. Adv. Funct. Mater..

[21] Bai J, Wang R, Li YP, Tang YY, Zeng QY (2016). A solar light driven dual photoelectrode photocatalytic fuel cell (PFC) for simultaneous wastewater treatment and electricity generation. J. Hazard. Mater..

[22] Greene LE, Law M, Goldberger J, Kim F, Johnson JC, Zhang YF, Saykally RJ, Yang PD (2003). Low-temperature wafer-scale production of ZnO nanowire arrays. Angew. Chem. Int. Ed..

[23] Lee JH, Leu IC, Hsu MC, Chung YW, Hon MH (2005). Fabrication of aligned TiO_2_ nanostructured arrays using a one-step templating solution approach. J. Phys. Chem. B.

[24] Jia QX, Iwashina K, Kudo A (2012). Facile fabrication of an efficient BiVO_4_ thin film electrode for water splitting under visible light irradiation. Proc. Natl. Acad. Sci..

[25] Mahajan VK, Misra M, Raja KS, Mohapatra SK (2008). Self-organized TiO_2_ nanotubular arrays for photoelectrochemical hydrogen generation: effect of crystallization and defect structures. J. Phys. D.

[26] Jin C, Zhang WG, Yao SW, Wang HZ (2012). Effect of heat-treatment process on the structure and photoelectric performance of TiO_2_ nanotube arrays. J. Inorg. Mater..

[27] Vitiello RP, Macak JM, Ghicov A, Tsuchiya H, Dick LFP (2006). N-doping of anodic TiO_2_ nanotubes using heat treatment in ammonia. Electrochem. Commun..

[28] Iwase A, Kudo A (2010). Photoelectrochemical water splitting using visible-light-responsive BiVO_4_ fine particles prepared in an aqueous acetic acid solution. J. Mater. Chem..

[29] Liu C, Ding Y, Wu W, Teng Y (2016). A simple and effective strategy to fast remove chromium (VI) and organic pollutant in photoelectrocatalytic process at low voltage. Chem. Eng. J..

[30] Zhao X, Zhang JJ, Qiao M, Liu HJ, Qu JH (2015). Enhanced photoelectrocatalytic decomposition of copper cyanide complexes and simultaneous recovery of copper with a Bi_2_MoO_6_ electrode under visible light by EDTA/K_4_P_2_O_7_. Environ. Sci. Technol..

[31] Liu L, Li R, Liu Y, Zhang J (2016). Simultaneous degradation of loxacin and recovery of Cu(II) by photoelectrocatalysis with highly ordered TiO_2_ nanotubes. J. Hazard. Mater..

[32] Munoz AG (2007). Semiconducting properties of self-organized TiO_2_ nanotubes. Electrochim. Acta.

[33] Kruger J, Plass R, Gratzel M, Cameron PJ, Peter LM (2003). Charge transport and back reaction in solid-state dye-sensitized solar cells: a study using intensity-modulated photovoltage and photocurrent spectroscopy. J. Phys. Chem. B.

