# Supplementary material for: BiVO4/TiO2(N2) Nanotubes Heterojunction Photoanode for Highly Efficient Photoelectrocatalytic Applications
Source: Nanomicro Lett. 2016 Nov 9;9(2):14. doi: 10.1007/s40820-016-0115-3 (PMC6223793; doi:10.1007/s40820-016-0115-3)
Supplement: Supplementary file 1 — Supplementary material 1 (DOCX 1529 kb) [file 40820_2016_115_MOESM1_ESM.docx]

**Supplementary support**


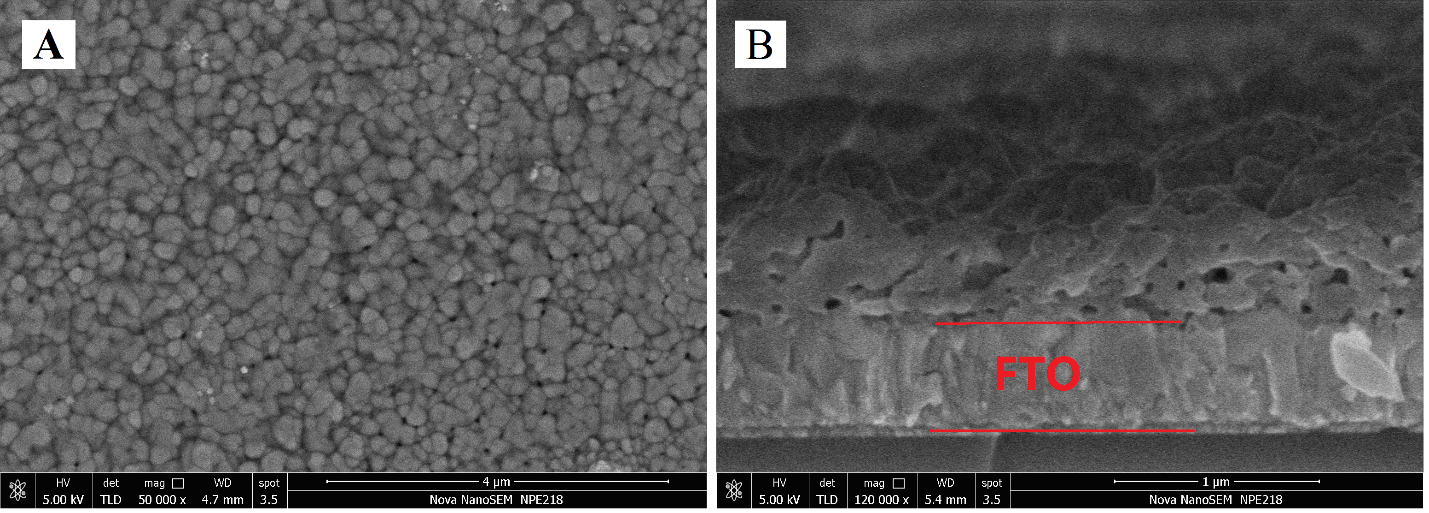


**Fig. S1** Top and cross-section SEM images of optimized BiVO_4_ photoandoe


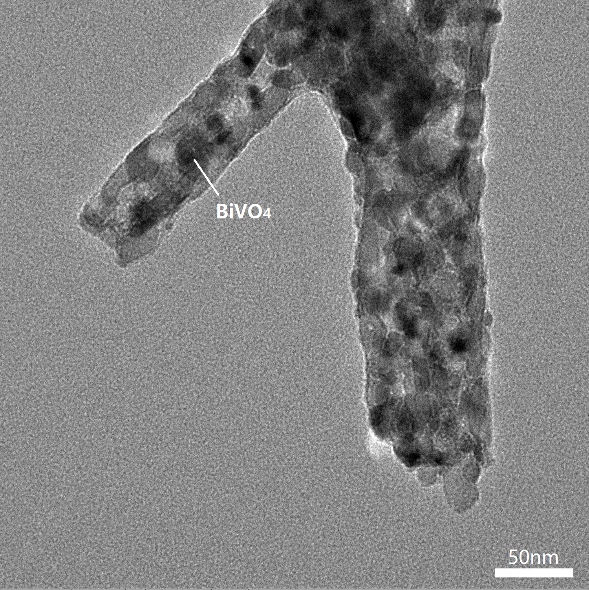


Figure S2 TEM images of BiVO_4_/TiO_2_(N_2_) NTs


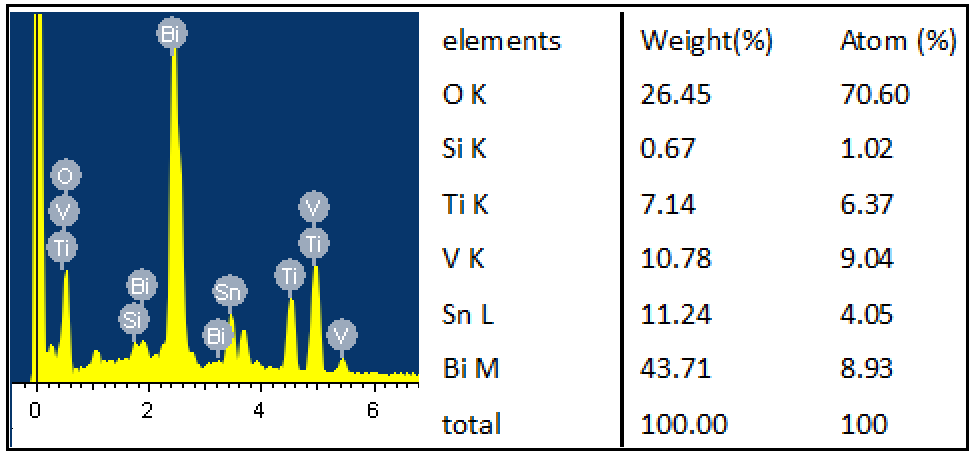


**Fig. S3** EDX spectrum of BiVO_4_/TiO_2_(N_2_) NTs





**Fig. S4** Photoelectrochemical cyclic voltammetry curve of the BiVO_4_/TiO_2_(N_2_) NTs under illumination of visible irradiation in 0.1 M Na_2_SO_4_ solution
